# P235: Incidence of nosocomial infection in long term institution and control measures

**DOI:** 10.1186/2047-2994-2-S1-P235

**Published:** 2013-06-20

**Authors:** ACDS Oliveira, S Scota, I França, AM Costa e Silva

**Affiliations:** 1 Educação Continuada e CCIH, Instituto de Infectologia Emílio Ribas, São Paulo, Brazil

## Introduction

The Premier Hospital specializes in the care of chronic patients with high dependency, especially elderly patients with chronic and degenerative diseases. It has 80 beds, with three units, one unit of semi-intensive care, emergency treatment and outpatient. Infections in the elderly are a major cause of morbidity, hospitalization and mortality and it is difficult to approach and diagnosis.

## Objective

To evaluate the rate of urinary tract infection in the institution after training the employees.

## Method

Data were collected from January to December 2012, the Commission of Infection Control. As the Brazilian statistics are under construction, we used for comparison, data from international incidence of infection/patient-days. Conducted training and guidance on hand hygiene and technique of bladder catheterization for employees of the institution.

## Results

Infection rates in 2011: Fee general Hospital Infection 8.73 infections/1000 patient-days and rate of urinary tract infection 3.35 infections/1000 patient-days. Infection rates in 2012: Fee general Hospital Infection 6.70 infections/1000 patient-days and rate of urinary tract infection: 2.37 infections/1000 patient-days.

## Discussion

It was established control measures to prevent Urinary Tract Infection. Held careful evaluation of the need for catheterization, training of nursing staff regarding the passage of the probe technique and care in maintenance, which also included simple precautions like using container unique to each patient to despise the urine. With these interventions decreased the rate of 3.35 infections/1000 Urinary Tract Infection patient-days in 2011 to 2.37 infections/1000 patient-days in 2012, achieving the value of international incidence data.

## Conclusion

We believe that training with continuous care in the insertion/maintenance and careful evaluation for catheterization, will achieve even lower rates of infection.

## Disclosure of interest

None declared

